# Rehabilitation of Neonatal Brachial Plexus Palsy: Integrative Literature Review

**DOI:** 10.3390/jcm8070980

**Published:** 2019-07-05

**Authors:** Fátima Frade, Juan Gómez-Salgado, Lia Jacobsohn, Fátima Florindo-Silva

**Affiliations:** 1 Nursing Department, Atlântica Health School, 2730-036 Barcarena, Portugal; 2 Department of Sociology, Social Work and Public Health, University of Huelva, 21007 Huelva, Spain; 3 Safety and Health Posgrade Program, Universidad Espíritu Santo, Samborondón, 091650 Guayaquil, Ecuador; 4 Physiotherapy Department, Atlântica Health School, 2730-036 Barcarena, Portugal; 5 Centro de Medicina de Reabilitação de Alcoitão, 2649-506 Alcabideche, Portugal; 6 Physiotherapy and Osteopathy Departments, Atlântica Health School, 2730-036 Barcarena, Portugal; 7 Serviço de Medicina Física e Reabilitação, do Hospital Dona Estefânia-Centro Hospitalar Universitário Lisboa Central, 1169-045 Lisboa, Portugal

**Keywords:** children, neonatal brachial plexus palsy, rehabilitation, patient safety, health care quality

## Abstract

This integrative literature review has been carried out with the aim of analyzing the scientific literature aimed at identifying and describing existing rehabilitation treatments/therapies for neonatal brachial plexus palsy (NBPP). NBPP is a frequent consequence of difficult birthing, and it impairs the function of the brachial plexus in newborns. This is why knowledge on rehabilitation strategies deserves special attention. The data collection was carried out in January 2019, in the EBSCOhost and BVS (Biblioteca Virtual em Saúde) platforms, in the CINAHL Complete, MEDLINE Complete, LILACS and PubMed databases. Thirteen articles were included in this integrative literature review, based on a literature search spanning title, abstract and full text, and considering the inclusion criteria. Two main treatments/therapies for NBPP rehabilitation were identified: conservative treatment and surgical treatment. Conservative treatment includes teamwork done by physiatrists, physiotherapists and occupational therapists. These professionals use rehabilitation techniques and resources in a complementary way, such as electrostimulation, botulinum toxin injection, immobilizing splints, and constraint induced movement therapy of the non-injured limb. Professionals and family members work jointly. Surgical treatment includes primary surgeries, indicated for children who do not present any type of spontaneous rehabilitation in the first three months of life; and secondary surgeries, recommended in children who after primary surgery have some limitation of injured limb function, or in children who have had some spontaneous recovery, yet still have significant functional deficits. Treatment options for NBPP are defined by clinical evaluation/type of injury, but regardless of the type of injury, it is unanimous that conservative treatment is always started as early as possible. It should be noted that there was no evidence in the literature of other types of rehabilitation and techniques used in clinical practice, such as preventive positioning of contractures and deformities, hydrotherapy/aquatic therapy, among others, so we consider there is a need for further studies at this level in this area.

## 1. Introduction

Neonatal Brachial Plexus Palsy (NBPP) is caused by traction of the brachial plexus during birth and can limit the function of the affected arm in various ways. It is the most common form of peripheral neuropathy, with an incidence rate of 0.5–2 cases per 1000 newborns in developed countries [1,2,3].

The causes associated with NBPP are macrosomia, breech/pelvic birth, diabetes in pregnancy, shoulder dystocia, small stature/cephalopelvic disproportion, primiparity, or a prolonged expulsion phase [4,5].

The clinical classification of NBPP is based on body structures, namely the complex of nerves that controls the affected finger, hand, arm, and shoulder muscles. In this sense, brachial plexus lesions are classified as “severing of the upper trunk” when the affected nerves are C5 and C6; “severing of the middle trunk” when the affected nerve is C7; and “severing of the lower trunk” when the affected nerves are C8 and T1. Finally, complete severing is considered when affecting C5–T1 nerves [6].

NBPP, in addition to being classified by considering the affected nerve roots, is also categorized according to the degree of lesion that affects the nerve and also to the function of the injured limb. According to the degree of lesion of the nerve, we can describe preganglionic and postganglionic avulsion injury (tearing near the dorsal root ganglion o near the spinal cord at an intraforaminal level, and tearing of the postganglionic nerve distant from the dorsal root ganglion and the spinal cord, respectively); neurotmesis lesion, also considered to be a severe injury of the nerve as there is a complete tearing of the axon and the connective tissue [7,8]; axonotmesis lesion, when there is an anatomic interruption of the axon but with no interruption or partial interruption of the connective tissue and the myelin; and, finally, stretching neuropraxic lesion without nerve rupture, implying a momentaneous physiological blockage of the nerve-axon connection, with spontaneous recovery. We must also consider neuroma lesion, which implies an interference of the injured nerve scar tissue that, when healing, does not allow the nervous impulse to the muscle.

Also termed Duchenne-Erb syndrome, upper brachial plexus palsy (C5–C6) is characterized by impaired abduction and external rotation of the shoulder and elbow flexion, while hand function is preserved. Also known as Dejerine-Klumpke syndrome, lower brachial plexus palsy (C7–T1) impairs hand and wrist function. In the case of a complete brachial plexus palsy (C5–T1), the function of the entire arm is impaired, presenting with a completely flaccid arm without sensitivity, and sometimes with ocular impairment. This combination of symptoms is known as Horner’s Syndrome [9,10,11,12,13].

With NBPP, prognosis and outcomes depend on the extent of the injury. The rehabilitation options depend on the type of injury and the regeneration evidenced by spontaneous recovery of the affected limb [12].

Rehabilitation treatment for neonatal brachial plexus palsy includes conservative treatment, started as soon as possible with passive movements, sensory stimuli and guidance to the child’s relatives, instead of surgical treatment, which implies surgical techniques, and is performed only after spontaneous recovery, usually at 3 months of age [14].

Thus, the aim of this integrative literature review is to analyze the scientific production so as to identify and describe the available treatments/therapies for the rehabilitation of neonatal brachial plexus palsy.

## 2. Experimental Section

This article is an integrative literature review. This type of review can cover both experimental and non-experimental research, providing a broader view of a given phenomenon [15].

In this review, we analyze and combine the data gathered through systematic searches, toward a further understanding of the subject of study: state of the art treatments for rehabilitating children with neonatal brachial plexus palsy.

This integrative literature review was structured based on a research theme and a research question, defined according to the PICo concept (Population (P); Interest Area (I), Context (Co)), on criteria for including and excluding articles, the choice of databases to search, and the selection and validation of descriptors on the DeCS (Descriptors in Health Sciences) and MeSH (Medical Subject Headings) websites. A Boolean conjunction was later used to search the databases, collect data, analyze the included studies, discuss and interpret the results and, finally, to draw a conclusion based on the main results. The research question was posed with resort to the PICo strategy: What are the available rehabilitation treatments/therapies for neonatal brachial plexus palsy?

The article search for this integrative literature review was performed during January 2019, using the EBSCOhost and BVS (Biblioteca Virtual em Saúde) platforms, and the CINAHL Complete, MEDLINE Complete, LILACS and PubMed databases. The following descriptors were eventually used: neonatal/obstetric brachial plexus palsy AND rehabilitation.

The inclusion criteria were availability (full-text articles), publication language (English, French, Spanish, and Portuguese), publication date (last five years), and all types of articles related to neonatal brachial plexus palsy. The references within the included articles were also considered. The exclusion criteria considered were articles that address brachial plexus palsy in adults (excluding articles on populations of 19 years of age or over this age), articles that address brachial plexus palsy non congenital and articles with costs of obtaining. Articles were selected by reading their title and abstract, and, whenever this proved insufficient, the full text of the article was read.

## 3. Results

**Figure 1:**
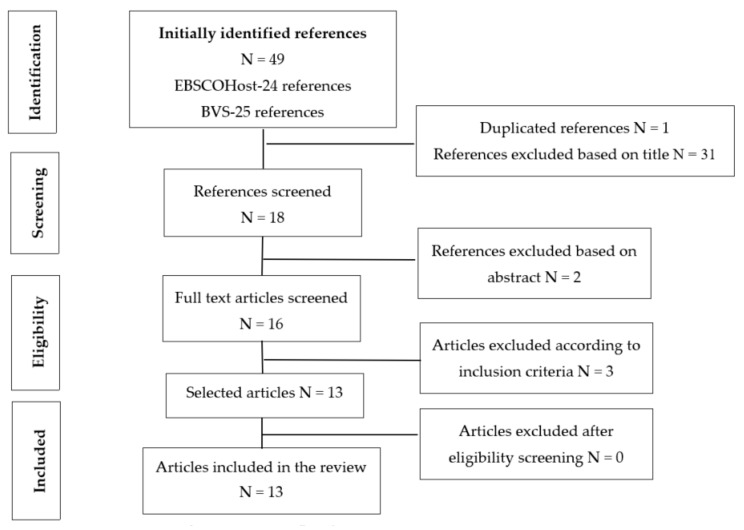
PRISMA flowchart.

Of the 49 initially identified articles, 13 were found to meet the inclusion criteria [4,16,17,18,19,20,21,22,23,24,25,26,27] after reading the title, abstract, and the full article. For a better understanding of the search strategy and selection of articles that constituted the sample for our integrative literature review, a PRISMA flow chart was built (Figure 1). Thus, 49 articles were then identified (24 in the FBSCOHost platform and 25 in the BVS platform). However, 1 article was excluded for finding a duplicate and 31 articles were also excluded after title search, resulting in 16 validated articles. After reading full texts and taking into account the inclusion criteria, 13 articles were selected as eligible and this is why our sample is made up of 13 articles.

Two of these articles were published in 2018 [19,22], one in 2017 [26], two in 2016 [16,25], three in 2015 [4,20,24], and five in 2014 [17,18,21,24,27].

The countries of origin were Argentina [16], the United States of America [19,22,23], Spain [26], the Netherlands [17], Canada [18], India [4], Cuba [21], Saudi Arabia [24,27], and Brazil [20,25].

The articles presented rehabilitation treatments/therapies for neonatal brachial plexus palsy, including (i) multidisciplinary conservative treatment [4,19,20,21,22] using complementary means or techniques such as electrostimulation, botulinum toxin injection, immobilizing splints and constraint induced movement therapy [4,19,20,22,23,26], and (ii) surgical treatment, which includes primary and secondary surgeries [16,20,22], where nerve transfer surgeries can use grafts of the median nerve, phrenic nerve, and ulnar nerve [24,25,27]. The criteria that define the choice for conservative or surgical treatment are debated by several authors [17,18,21,22,27] (Table 1).

## 4. Discussion

### 4.1. Multidisciplinary Conservative Treatment

Conservative treatment of neonatal brachial plexus palsy involves early diagnosis and follow-up, if possible, within two to three weeks after the child’s birth [21]. Conservative treatment should involve a multidisciplinary team, composed of physiatrists, clinical neurophysiologist, neurosurgeons, occupational therapists, and physiotherapists. The treatment administered by the physiotherapist and occupational therapist involves smooth joint movements and sensory stimulation, such as passive/active mobilization exercises, stretches, tactile stimulation with different textures, vibration and brushing techniques to promote sensory ability in the injured limb, and bimanual activities. Sensory stimulation is as important as motor stimulation and can consist in suckling any finger on the injured limb and stimulating the skin with different textures, temperatures, and vibrations [4,19,20,21,22]. Electrical stimulation/electrostimulation is a complementary means or technique used in conservative therapies for the rehabilitation of brachial plexus palsy [4,19], that promotes gaining muscle tone/strength on the affected muscles, and significant improvements in the mobility of the injured limb. These therapies aim to ensure the conditions needed for the functional recovery of the limb following nerve regeneration, which implies the prevention of muscle shrinkage, sagging, joint deformities, and muscle contractures [20,21]. Both therapies play a key role in the rehabilitation of neonatal brachial plexus palsy, but it is essential to involve parents in the rehabilitation program, so that professionals and family members work jointly. Therapy should be administered several times a week and, at home, as frequently as possible, for example at each meal or with every nappy change [20,21]. Most studies reveal that conservative treatment performed by therapists significantly reduces injuries, removing the need for surgical intervention [28,29].

There are different tools that are used as means and/or complementary techniques to the conservative/surgical treatment of neonatal brachial plexus palsy, such as electrostimulation, botulinum toxin injection, thermoplastic splints, posterior and anterior temporary splints (for physiological positioning, facilitating functional motor function, and preventing vicious postures. Anterior and posterior fist or hand splints control and prevent, at the fist level, extreme ulnar flexion and deviation. Anterior splints can simultaneously control thumb adduction, and posterior splints allow more freedom of the child’s palm), and constraint induced movement therapy [4,19,20,22,23,26].

Electrostimulation is commonly used to increase muscle strength, with the aim of promoting functional muscle recovery after nerve injury. It inhibits muscle atrophy during the reinnervation period, and accelerates nerve regeneration, resulting in improved muscle strength and range of motion in the injured limb [4,19,22]. The benefits of electrostimulation in the recovery of neonatal brachial plexus palsy as a complement to conservative/surgical treatment are evident [30].

Injection of botulinum toxin into healthy antagonist muscles has proven effective in the treatment of muscle imbalances, co-contractions, and muscle contractures in children with neonatal brachial plexus palsy. The aim of this complementary treatment option is to balance strength and to allow the affected muscles to develop, by adapting the movement pattern to the ongoing nerve recovery [26]. Studies on the benefits of using botulinum toxin for treating neonatal brachial plexus palsy demonstrate benefits for the elbow function, with improved flexion and supination [31].

The use of temporary immobilizing splints is indicated for children with impaired wrist function, which can help improve hand function and prevent wrist drop, thus promoting wrist extension. Some splints are used during sleep, and other more functional ones are used during awake time activities [20,21].

Constraint induced movement therapy demonstrates that performing activities at home for one hour a day can improve mobility, functional capacity, speed, range of motion, and hand manipulation ability [23]. Other studies reveal the effectiveness of movement therapy, leading to improvements in mobility, increased predisposition to use the injured limb, and frequency of use [32,33].

### 4.2. Surgical Treatment

Surgical nerve reconstruction may be necessary for rehabilitating patients with neonatal brachial plexus palsy, especially children who do not show spontaneous recovery during the first months of life [17]. However, conservative treatment should be favored whenever possible [16].

When surgical intervention is required, both primary and secondary microsurgeries are available. Primary microsurgery techniques include recession and reconstruction of the neuroma, neurolysis, and nerve transfer [16,20,22]. Studies reveal that, as a primary surgery for neonatal brachial plexus palsy, neurolysis combined with nerve transfer produces good results [34].

Nerve transfer surgeries reconnect nerves that have less important roles or are redundant with the target nerve, without innervation [22].

In situations where the lesion affects the suprascapular nerve, shoulder function is impaired (abduction and external rotation). Grafts extracted from the proximal C5 root stump or the accessory nerve are often used to reconstruct the suprascapular nerve. The use of the phrenic nerve has also been shown to provide a similar level of recovery to the use of the median nerve, increasing the number of graft options available to recover suprascapular nerve function [24].

When the C5–C6 nerve roots are affected, i.e., in Erb’s palsy, affecting shoulder abduction and external rotation, elbow flexion, and forearm supination, and when there is no evidence of spontaneous recovery, surgery is a valid treatment option. The Oberlin’s procedure involves the transfer of the ulnar nerve to the cutaneous nerve and is an effective way of recovering the elbow function, improving elbow flexion and leading to increased functional use of the affected limb [25]. Other studies corroborate the positive results in the recovery of the biceps function obtained with the Oberlin’s procedure [35,36].

Another alternative for treating Erb’s palsy is to extract a graft from the median nerve and use it to reconstruct the biceps nerve, which has been shown to improve elbow flexion [27]. The use of the ulnar or median nerves as grafts for treating the biceps are viable options, not only because of the evidence supporting the recovery of the biceps function, but also due to the proximity of the biceps nerve [37].

Secondary surgery involves tendon transfer, arthrodesis, or osteotomies, and may be an option for children who have partially recovered after primary surgery but still show some deficits deemed treatable, or for children who experience spontaneous recovery but still show some functional deficits. In summary, primary surgery is the initial treatment option for children who do not experience spontaneous recovery, and secondary surgery aims to promote the functional improvement of the limbs [16]. Other authors report that secondary surgery may include procedures to improve external shoulder rotation, such as the Hoffer’s procedure, and the Steindler flexorplasty to improve elbow flexion. Secondary surgery is also used to improve hand function, but relevant studies suggest that improving hand function is a great challenge, and the results of these surgeries are merely palliative in most cases [38,39,40].

In general, with regard to surgical treatment, the authors argue that primary surgery includes surgical procedures involving nerve transfer, and the ulnar, median and phrenic nerves are used as grafts/donors in this type of surgery. Secondary surgeries are used in patients who, after primary surgery, have reached a certain level of recovery but still show some deficits considered treatable, or in patients who have not undergone primary surgery and show some type of spontaneous recovery but still have deficits.

### 4.3. Criteria for Conservative/Surgical Treatment

Criteria for opting between conservative treatment or surgical treatment for neonatal brachial plexus palsy are not consensual. There is no scientific evidence favoring surgical treatment/nerve reconstruction over conservative treatment. Children who do not show spontaneous recovery during the first months of life are considered suitable recipients of reconstructive nerve surgery [17].

Some studies defend that primary surgery (neuroma excision or nerve graft) is indicated for children who do not have biceps function (elbow flexion against gravity) at the age of 3 months. If at this age there is evidence of nerve root avulsion, surgery is indicated. Some authors defend that the decision to operate can be postponed until 5–6 months if there is no biceps function at the age of 3 months, but some level of shoulder recovery is observed [18,21].

Some studies defend that if there is some level of recovery of the biceps function at the age of 3 months, the situation can be reassessed between the age of six and nine months to assess whether there is need for surgical intervention [22]. Other authors corroborate the influence of biceps recovery in opting for conservative/surgical treatment, stating that biceps recovery before the age of three months is a predictor of complete or near-complete shoulder recovery. The exact moment to decide on nerve reconstruction is difficult to identify, but a possible range is established between the child’s third and sixth month of life [41,42].

Neonatal brachial plexus palsy is complex, present with many different severity levels and prognoses, as well as reinnervation and recovery patterns, which are unpredictable factors that make it harder to define rigorous criteria for reconstructive surgery [18]. Several scientific studies reveal how difficult it is to opt for surgical treatment of neonatal brachial plexus palsy. For severe lesions, with avulsion and rupture of the nerve roots, the neurological prognosis without surgical intervention is poor, justifying surgical treatment. Another strong indicator for surgery is decreased hand function without spontaneous recovery. For children that present with partial lesions at the C5–C6 or C5–C6–C7 level, surgery is a "grey area", as different levels of spontaneous recovery can take place, leading to the decision for surgery at different stages during the child’s life, namely at 3 months of age, 5–6 months, or up to 9 months [43,44].

Surgical treatment is not a consensual option among the different authors. Some authors defend the surgical intervention at the age of three months if there is no spontaneous recovery or if there is evidence of nerve root. Others defend the evaluation surgery at the age of 5–6 months, or at 6–9 months in children who, despite lacking biceps function at three months of age, experienced at this time some spontaneous recovery of the injured limb. An early rehabilitation treatment based on intensive multidisciplinary conservative treatment can lead to favorable functional outcomes in children whose biceps recover spontaneously between the ages of 3 and 6 months. When there is no spontaneous recovery or complete paralysis of the limb, the most widely prescribed treatment is the surgical treatment.

## 5. Conclusions

In the rehabilitation of NBPP there are two options of therapies or treatments: the conventional/conservative treatment and the surgical treatment. The conventional/conservative multidisciplinary treatment, which includes intensive physiotherapy/occupational therapy sessions, using in a complementary way, means and or techniques such as electrostimulation, immobilizing splints, constraint induced movement therapy, the work of joint action with families, and/or botulinum toxin injection; and the surgical treatment that includes primary and secondary surgeries, respectively the first ones are indicated for children who do not have any type of spontaneous rehabilitation in the first three months of life, and the later are an option for ex-post treatment for children who still present functional limitations or significant functional deficits of the injured limb, after spontaneous recovery or in children who were subjected initially to primary surgery. There is unanimous agreement that early conservative treatment is the main treatment option for the rehabilitation of neonatal brachial plexus palsy. Whatever the type of lesion, it is generally expected that clinical development/recovery will progressively help defining the diagnosis, thus facilitating decision making to maintain the conservative treatment or opt for surgical treatment, restarting the intensive conservative treatment after the surgeries.

Despite the important conclusions that this integrative literature revision allowed us to identify in relation to the rehabilitation treatment options of NBPP, we also noticed that there is no current scientific evidence on some rehabilitation means/techniques used in conservative treatments, that is, some positioning of the limbs (like external rotation of the shoulder and forearm to prevent contractures and deformities (glenohumeral dysplasia); the use of kinesiology tapes; the use of weight shift on the injured limb (at key stages of the child’s development), and hydrotherapy from the age of 6 months). Due to this multiplicity, we identified the need for further research on these types of means/techniques.

## Figures and Tables

**Table 1:** Description of the results.

Author(s), Year, Location	Population	Aims	Methods
Socolovsky, M; et al. 2016, Argentina [16]	Articles about primary and secondary surgery.	Identify and describe the primary and secondary surgeries available for the rehabilitation of obstetric brachial plexus palsy.	Literature Review.
**Results:** Primary surgery may be unnecessary if there is spontaneous recovery of the biceps before the age of 3 months. Authors defend a wider decision window for primary surgery ranging from 3 to 9 months after birth, avoiding unnecessary surgeries and favoring conservative treatment in the event of any recovery in elbow or shoulder function. There are three primary microsurgery techniques: (1) recession and reconstruction of the neuroma using interposed grafts, (2) neurolysis, and (3) nerve transfer. Secondary surgical procedures involve tendon transfer, arthrodesis, or osteotomies, and can be used in patients who have partially recovered after primary surgery, or in patients who have not undergone primary surgery and experience some form of spontaneous recovery, but still have some deficits. Secondary surgery targets the (1) shoulder, (2) elbow, and (3) hand.			
**Author(s), Year, Location**	**Population**	**Aims**	**Method**
Pondaag, W; Malessy, M. 2014, Netherlands [17]	Nine articles	Analyze and describe scientific evidence on conservative rehabilitation and surgical rehabilitation of obstetric brachial plexus palsy.	Systematic Literature Review
**Results:** The studies analyzed in this systematic review of the literature do not present scientific evidence favoring surgical treatment/nerve reconstruction over conservative treatment, where spontaneous recovery occurs. Most of the analyzed studies reveal surgical reconstruction of the nerves was performed in children who showed no spontaneous recovery during the first months of life.			
**Author(s), Year, Location**	**Population**	**Aims**	**Methods**
Bade, S; et al. 2014, Canada [18]	Seventeen patients with obstetric brachial plexus palsy	Explore situations where reconstructive surgery is indicated for obstetric brachial plexus palsy.	Exploratory Study
**Results:** There is no consensus on indication and length of surgical treatment for obstetric brachial plexus palsy. It is advocated that excision of the neuroma or nerve grafting is indicated for children who do not have biceps function at the age of 3 months. Flexion and extension of the elbow combined with wrist extension, extension of the fingers, and thumb extension improve the prognosis of spontaneous recovery. Surgery is indicated if at 3 months of age there is evidence of avulsion of the nerve roots. The study shows that the biceps of children can recover at 5 months, but surgery is only counter-indicated at 3 months if upper function (shoulder) is partially recovered, especially the external rotation of the shoulder.			
**Author(s), Year, Location**	**Population**	**Aims**	**Methods**
Justice, D; Awori, J. 2018, USA [19]	Four articles	Evaluate the efficacy of neuromuscular electrostimulation in restoring movement and function in neonatal brachial plexus palsy.	Literature Review
**Results:** Physiotherapists and occupational therapists often use neuromuscular electrostimulation to treat decreased muscle strength. Neuromuscular electrostimulation treatment involves using electrodes to deliver an electrical current to a muscle without nervous activity, with the aim of promoting functional recovery of the muscle after nerve injury. Electrostimulation is used concomitantly with exercise, massage, splinting, constraint induced movement therapy, and Ayurvedic therapy. Studies have shown that the use of electrostimulation inhibits muscle atrophy during the reinnervation period, and accelerates nerve regeneration, so an improvement in muscle strength and range of motion is associated with electrostimulation.			
**Author(s), Year, Location**	**Population**	**Aims**	**Methods**
Jeyanthi, S; 2015, India [4]	One child with obstetric brachial plexus palsy.	Describe the benefits of electrostimulation in improving motor performance and functional performance in a child with obstetric brachial plexus injury.	Case study.
**Results:** Conventional treatment of obstetric brachial plexus injury advocates early diagnosis and follow-up accompanied by physiotherapy and occupational therapy. In these therapies, which include passive mobilization, stretches, use of anterior and posterior splints to prevent deformities, especially during sleep, and early motor training to encourage efficient movements. Tactile stimulation is provided through different materials of different textures; vibration and brushing techniques to increase sensory awareness of the affected arm; bimanual activities to prevent limb disuse; strength and coordination exercises throughout the child’s growth and development stage; and day-to-day activities that promote fine motor control. Electrostimulation is a technique used by these therapists with the aim of promoting rehabilitation, preventing muscle atrophy, promoting nerve regeneration, and increasing motor performance and joint manipulation, namely in the shoulder, elbow, and wrist, as well as increasing awareness of the limb. This study concludes that, as a complement to conventional therapies, electrical stimulation of nervous branches helps solve functional limb impairment and promotes active movement gains, especially in the biceps, and wrist and finger extensors.			
**Author(s), Year, Location**	**Population**	**Aims**	**Methods**
Otto, HC; et al. 2015, Brazil [20]	Articles on rehabilitation of neonatal brachial plexus palsy in infants	Describe current practices for rehabilitating lactating infants with neonatal brachial plexus palsy, covering conservative and surgical therapy.	Literature Review
**Results:** Rehabilitation of neonatal brachial plexus palsy may include conventional treatment through therapies such as physiotherapy and occupational therapy, or surgery. The indication for surgery is not consensual. Some authors defend that surgery is indicated if there is no evidence of spontaneous recovery, especially of the biceps function in children up to the age of 3 months. Others defend that this assessment can be made later, up to the age of 6 months. This assessment not only covers elbow flexion, but also shoulder abduction, and elbow, wrist, and finger extension. In conventional treatment, physiotherapy and occupational therapy are of great importance, but it is essential to involve parents in the rehabilitation program, so that professionals and family members work jointly. A passive range of motion exercises are fundamental for preventing muscle contractions and should be done several times a day. It is important to include them in all day-to-day activities, such as with every nappy change, and at each meal (bottle/breast). As the child grows, develops, and gains intentional voluntary control and awareness of his/her body, it is important to promote activities that stimulate the affected limb so as to prevent apraxia. One possible strategy is to encourage bimanual activities. In children with compromised wrist extension, the use of temporary, posterior and anterior immobilizing splints can help improve hand function and prevent wrist drop. Splints are best used during sleep, so that the hand can be used during awake time activities. Excessive reinnervation of active muscles can result in co-contractions and contractures, which can be treated with botulinum toxin, allowing gains in mobility and range of motion. Regarding surgery, there are three approaches: neurolysis, nerve grafts, and nerve transfers.			
**Author(s), Year, Location**	**Population**	**Aims**	**Methods**
Yanes, SVL; et al. 2014, Cuba [21]	Theoretical references that address frequency, risk factors, aetiology, anatomy, and pathophysiology, lesion types, prognosis, and treatments.	Describe the clinical elements related to obstetric brachial plexus palsy and present the different rehabilitation strategies.	Literature Review
**Results:** Currently, conservative treatment is recommended, i.e., physiotherapy and occupational therapy, engaging parents as important actors in the child’s rehabilitation. Authors defend that conservative treatment must be applied as early as possible (2 to 3 weeks after birth) through gentle joint movements and sensory stimulation. The aim of the therapy is to ensure the necessary conditions for functional recovery following nerve regeneration, which implies the prevention of muscle shrinkage, sagging, and joint deformities through activities adapted to the child’s development. The therapy should be administered several times a week, and at home, as frequently as possible, for example at each meal or with every nappy change. Sensory stimulation is as important as motor stimulation and can consist in suckling any finger on the injured limb and stimulating the skin with different textures, temperatures, and vibrations. The use of thermoplastic splints may be necessary to maintain wrist extension. Electrostimulation is also used in conservative treatment. As the child grows, rehabilitation remains important for integrating the limb into the body structure through integration activities and postural re-education of the upper limb and the chest. Exercises that target the recovery of muscle strength are important, even if neurological and muscle functions are not fully recovered. Surgery presents itself as an alternative for conservative treatment, with some authors defending that children who have not recovered their biceps function at the age of three months must undergo surgery. Others prefer to wait up to the age of six months if nerve root avulsion is not suspected.			
**Author(s), Year, Location**	**Population**	**Aims**	**Methods**
Smith, B; et al. 2018, USA [22]	Articles on rehabilitation of neonatal brachial plexus palsy in infants.	Provide an update on the rehabilitation of neonatal brachial plexus palsy to replace old beliefs with new paradigms.	Literature Review
**Results:** The management of neonatal brachial plexus palsy has changed considerably in the last 25 years, leading to a paradigm shift. The old indication to immobilize the arm after diagnosis of the lesion is completely outdated. Currently, after early diagnosis of the lesion, the child is expected to undergo conservative treatment with physiotherapy and occupational therapy as soon as possible. The aim of these therapies is to promote a normal range of motion, the use of the affected limb, to strengthen muscles and develop independence. The child’s family should be involved in the rehabilitation of neonatal brachial plexus palsy and trained on exercises that can be performed at home. Various techniques are used in these therapies: stretches, passive and active movements, muscle strengthening, and use of kinesiology tapes and electrostimulation, among others. As an alternative to conservative treatment, studies performed at the University of Toronto indicate that there is a surgical option for children without bicep function at the age of 3 months. If there is any evidence of recovery at the age of three months, the situation is reassessed between the age of 6 and 9 months to assess whether there is need for surgical intervention. Assessment at these stages indicate the need of early follow-up for children with obstetric brachial plexus palsy. Surgical options for these lesions involve nerve grafts, nerve transfer, or a combination of both. The nerve graft can be extracted from the nerve itself (autologous graft). Nerve transfers reconnect a nerve from a muscle with redundant function to the target without innervations.			
**Author(s), Year, Location**	**Population**	**Aims**	**Methods**
Brown, SH; et al. 2015, USA [23]	A 17-year-old female patient with neonatal brachial plexus palsy.	Determine the efficacy of a therapy program administered at home, where movement of the healthy arm of a 17-year-old adolescent with neonatal brachial plexus palsy was constrained.	Case Study
**Results:** This therapy consists in constraining movement of the non-injured arm to stimulate movement of the injured arm through active exercises that promote mobility, range of motion, and strength. This case study demonstrates that constraining movement therapy of the non-injured arm, administered at home through activities performed one hour per day, can improve mobility, functional capacity, speed, range of motion, and hand manipulation ability in a teenager with neonatal brachial plexus palsy.			
**Author(s), Year, Location**	**Population**	**Aims**	**Methods**
Al-Qattan, MM; El-Sayed, AAF. 2014, Saudi Arabia [24]	A total of 15 infants underwent phrenic nerve transfer to reconstruct the suprascapular nerve, 15 lactating infants underwent accessory nerve transfer to reconstruct the suprascapular nerve, and the potential to improve external shoulder rotation was studied.	Verify if using a phrenic nerve graft to reconstruct the suprascapular nerve in case of an obstetric brachial plexus palsy contributes to improving external shoulder rotation-	Retrospective Study
**Results:** Surgery is one of the rehabilitation options for obstetric brachial plexus palsy, and there are several types of surgical procedures such as nerve grafting, among others. When the lesion affects the suprascapular nerve, shoulder function is impaired, especially abduction and external rotation. Grafts used to reconstruct the suprascapular nerve are often extracted from the proximal C5 root stump or the accessory nerve. This study introduces a communicating branch from the phrenic nerve as a potential donor for rebuilding the suprascapular nerve, when available and a suitable size. The study showed no significant improvement in the recovery of the shoulder’s external rotation when the phrenic nerve or the accessory nerve were used as grafts. Therefore, we can assume that the communicating branch from the phrenic nerve can be considered an option to rebuild the suprascapular nerve.			
**Author(s), Year, Location**	**Population**	**Aims**	**Methods**
Figueiredo, et al; 2016, Brazil. [25]	A total of 13 children between the ages of 9 and 15 months were treated according to the Oberlin’s Procedure.	Describe the results of ulnar nerve transfer to the cutaneous nerve (Oberlin’s method) in the improvement of elbow flexion, in children with obstetric brachial plexus palsy at the C5–C6 level.	Retrospective Study
**Results:** Obstetric brachial plexus palsy may involve neuropraxia or nerve root avulsion. The C5–C6 roots are frequently affected in obstetric brachial plexus palsy, compromising shoulder abduction and external rotation, elbow flexion, and forearm supination. This type of lesion is known as Erb’s palsy. Elbow flexion is a fundamental aspect of arm function, and when absent in the affected arm, one of the treatment options is surgery. The Oberlin’s procedure involves the transfer of the ulnar nerve to the cutaneous nerve, with the aim of improving elbow flexion. In this study, thirteen children underwent the abovementioned procedure. After surgery, the arm was immobilized with the shoulder in adduction and the elbow in flexion to the right. Physiotherapy and occupational therapy were then resumed, with specific activities to develop functionality, as well as active and passive mobilization. To evaluate the efficacy of the procedure, elbow flexion, supination of the forearm, and limb function were assessed, as was the nervous function of the donor during wrist flexion. The results reveal that the Oberlin’s procedure significantly improves elbow flexion and functional use of the affected limb, without any loss of hand function, namely wrist flexion, because the ulnar nerve was used as a donor. No conclusions about supination can be drawn from the results. Nonetheless, the Oberlin’s procedure is an effective option for recovering elbow function and should be consider alongside other procedures to restore shoulder function.			
**Author(s), Year, Location**	**Population**	**Aims**	**Methods**
García Ron, A; et al. 2017, Spain [26]	Fifteen cases of newborns with obstetric brachial plexus palsy.	Describe the treatment of an obstetric brachial plexus palsy with ultrasound-guided botulinum toxin infiltration.	Prospective, Descriptive Study
**Results:** The severity and extent of the obstetric brachial plexus palsy determine recovery and sequelae. Some of these lesions recover spontaneously during the first 6 to 8 weeks of life. However, there are more severe cases that leave permanent motor sequelae and that are greatly disabling for limiting movement, strength and volume in the affected muscle. Permanent functional limitation is frequently found in the more severe lesions, particularly affecting the muscles involved in external shoulder rotation and abduction, elbow flexion, forearm supination, and wrist extension. The weakened state of these muscles leads to a great imbalance of strength in relation to the healthy muscles. These persisting muscle imbalances limit the strength and movement of the affected limb, leading to muscle contractures and bone deformities, particularly in the shoulder. Treatment with botulinum toxin has shown efficacy in the treatment of muscle imbalances, co-contractions, and muscle contractures. It is used to treat children with obstetric brachial plexus palsy, weakening healthy antagonistic muscles with the aim of balancing strength, and allowing the affected muscles to develop. Strength and functional improvement are addressed through occupational therapy and physiotherapy, with movement patterns suited to the ongoing nerve recovery. In this study, botulinum toxin infiltrations were started between the child’s 6th and 18th month of life, with an average of three infiltrations per child. The most frequently infiltrated muscles were the subscapular, the pectoralis major, the latissimus dorsi, and the pronator teres. Results appear 2 to 3 weeks after infiltration, and the effect has an average duration of 4 months. In this study, botulinum toxin infiltration managed to spare 3 children from undergoing surgery. All children improved functionality of the limb and abnormal postures: improvement was observed both in the shoulder joint and the elbow joint. In conclusion, it is important to use infiltration with botulinum toxin as adjuvant treatment to physiotherapy and/or surgical treatment in moderate/severe obstetric brachial plexus palsy.			
**Author(s), Year, Location**	**Population**	**Aims**	**Methods**
Al-Qattan, MM; Al-Kharfy, TM. 2014, Saudi Arabia [27]	Ten cases of children with upper (C5–C6) obstetric brachial plexus palsy, with little or no late recovery of elbow flexion.	To describe the results of median nerve transfer to the biceps nerve, with the aim of reinnervating the biceps of children with obstetric brachial plexus palsy at the C5–C6 level, known as Erb’s palsy.	Descriptive Study
**Results:** Surgical treatment is indicated in the absence of spontaneous recovery of elbow flexion in children with obstetric brachial plexus palsy at the C5–C6 level (Erb’s paralysis), where the biceps nerve is grafted. Postoperative care includes the immobilization of the affected arm for three weeks before intensive physiotherapy is restarted. This study describes the recovery of ten children with Erb’s palsy, who underwent reconstructive surgery of the biceps nerve, using the median nerve as a donor. Reinnervation of the biceps nerve using the median nerve leads to improved elbow flexion.			
